# Pharyngocutaneous fistula following total laryngectomy

**DOI:** 10.5935/1808-8694.20120040

**Published:** 2015-10-20

**Authors:** Felipe Toyama Aires, Rogério Aparecido Dedivitis, Mario Augusto Ferrari de Castro, Daniel Araki Ribeiro, Claudio Roberto Cernea, Lenine Garcia Brandão

**Affiliations:** a Medical Student at the Lusíada University Center in Santos - SP (Medical Student at the Lusíada University Center in Santos - SP); b Senior Associate Professor, Supervisor of the Larynx Group in the Department of Head and Neck Surgery in the Hospital of the Medical School of the University of São Paulo (MD); c MSc in General Practice by the Lusíada University Center UNILUS (Assisting Physician in the HNS services of the Ana Costa Hospital and the Santa Casa da Misericórdia de Santos, Santos - SP); d Adjunct Professor and Senior Associate Professor in the Department of Biosciences of the Federal University of São Paulo - UNIFESP - Baixada Santista Campus, Santos; e Associate Professor in the Department of Head and Neck Surgery of the Medical School of the University of São Paulo, São Paulo (Associate Professor in the Department of Head and Neck Surgery of the Medical School of the University of São Paulo, São Paulo); f Professor in the Department of Head and Neck Surgery of the Medical School of the University of São Paulo, São Paulo (Professor in the Department of Head and Neck Surgery of the Medical School of the University of São Paulo, São Paulo). Head and Neck Departments - Ana costa and Irmandade da Santa Casa de Misericórdia de Santos - Hospitals. Brazil

**Keywords:** carcinoma, squamous cell, cutaneous fistula, laryngeal neoplasms, laryngectomy, postoperative complications

## Abstract

Pharyngocutaneous fistula (PCF) is the most common complication after total laryngectomy.

**Objectives:**

To establish the incidence of this complication and to analyze the predisposing factors.

**Method:**

This is a cross-sectional study of a historical cohort including 94 patients who underwent total laryngectomy. The following aspects were correlated to the occurrence of PCF: gender, age, tumor site, TNM staging, type of neck dissection, previous radiation therapy, previous tracheotomy, and use of stapler for pharyngeal closure. The following were considered in PCF cases: the day into postoperative care when the fistula was diagnosed, duration of occurrence, and proposed treatment.

**Results:**

Twenty (21.3%) patients had PCF. The incidence of PCF was statistically higher in T4 tumors when compared to T2 and T3 neoplasms (*p* = 0.03). The other analyzed correlations were not statistically significant. However, 40.9% of the patients submitted to tracheostomy previously had fistulae, against 21.1% of the patients not submitted to this procedure.

**Conclusion:**

Advanced primary tumor staging is correlated with higher incidences of PCF.

## INTRODUCTION

Pharyngocutaneous fistula (PCF) is the most commonly reported postoperative complication in total laryngectomy patients. PCF significantly increases morbidity, length of hospitalization, and cost of care, in addition to delaying the start of indicated adjuvant therapy^1^. Salivary fistulae also predispose patients to neck large vessel injuries and cause considerable discomfort as patients have to be fed through naso-gastric tubes^2^. The literature reports PCF incidence rates ranging from 3% to 65%^3^, ^4^, ^5^.

Many factors related to the incidence of PCF such as age, gender, smoking and alcohol consumption during the disease, liver function, anemia, previous radiotherapy, previous tracheostomy, neck dissection, comorbidities (diabetes, decompensated congestive heart failure, malnutrition, chronic bronchitis) and even postoperative vomiting have been the topic of controversy. The factors known to be associated with increased incidence are inadequate surgery, and hematoma of the surgical wound. In cases of late fistulization, second primary tumor of the piriform sinus must be considered^6^, ^7^, ^8^, ^9^, ^10^.

This paper aimed to establish the incidence of this complication and analyze predisposing factors in a series of patients seen at a specialized care center.

## METHOD

This study was approved by the Research Ethics Committee of the institution in which it was carried out. This retrospective cohort study included the analysis of the charts of patients diagnosed with laryngeal squamous cell carcinoma (SCC) submitted to total laryngectomy in two tertiary care hospitals. All procedures were performed by two surgeons, who followed all the patients and tracked the postoperative onset of PCF.

From 1996 to 2011, 94 patients underwent total laryngectomy. These patients were studied retrospectively for the occurrence of PCF. The following variables were considered: gender, age, primary tumor site, primary tumor TNM staging, type of neck dissection, previous radiotherapy, previous tracheostomy, and the use of manual stitches or stapler to close the pharynx. Total laryngectomy patients diagnosed with SCC by biopsy were enrolled in the study. Indication of pharyngeal flap closure was considered as a criterion for exclusion.

As a routine, patients were given a prophylactic combination of antibiotics amikacin and clindamycin during the first day after surgery, with the first dosage given at the time of anesthesia induction. Subjects underwent primary closure of the pharyngeal remnant without flap rotation. Flap surgery was a criterion for subject exclusion.

Two types of pharyngeal closure procedures were performed: manual closure using T-shaped stitches with polyglactin 3-0; and closure with a 75 mm TCL75 Ethicon® linear stapler. The stapler was first used in 2003. As a routine, a primary tracheoe-sophageal fistula was not created during surgery to place the voice prosthesis. The prosthesis was put in place three months after surgery.

In PCF cases, the day of PCF onset into postoperative care, duration, length of hospitalization, therapy, and patient evolution were considered. Diagnosis was done through the observation of saliva secretion in the continuous suction tube. Patients were given methylene blue to facilitate fistula visualization.

Nasoenteral feeding tubes were placed on the first day after surgery. Oral feeding was initiated on day 10 after surgery in PCF-negative patients. Late fistulization was not observed in patients cleared for oral feeding on day 10 after surgery.

Dichotomic data univariate analysis was performed using a 2x2 matrix and the chi-square test. Continuous data sets were analyzed by the difference between mean values and their respective standard deviations. A *p*-value of 0.05 was adopted.

## RESULTS

Ninety-four laryngeal SCC patients submitted to total laryngectomy were analyzed. Eighty-seven were males (92.5%). Subject age ranged from 36 to 89 years, and mean age was 62.0 ± 11.2 years.

The global incidence of PCF was 21.3% (20 subjects). In the group with PCF, 85% were treated conservatively with antibiotics, enteral feeding, local hygiene, and neck compression. The three patients treated surgically were offered the myocutaneous flap procedure.

The outcomes concerning the analyzed predisposing factors can be seen on Table 1.

**Table 1:** Correlation between variables and incidence of pharyngocutaneous fstula.

Variable	PCF n = 20	No PCFn = 74	*p*
Gender			
Male	19	68	0.59
Female	1	6	
Age			
< 60	4	24	0.24
> 60	16	50	
T-stage			
T2/T3	11	60	
T4	9	14	0.03
Tracheostomy			
Yes	9	22	0.22
No	11	52	
Previous chemoradiotherapy			
Yes	8	24	0.54
No	12	50	
Neck disection			
Radical	13	46	0.81
Selective	7	28	
Pharyngeal closure			
Manual	14	60	0.32
Stapler	6	14	

Seven patients had T2 tumors, 64 had T3 disease, and 23 had T4 neoplasms. Patients diagnosed with advanced disease (T4) were at increased risk of PCF by an additional 24% when compared to subjects with T2-T3 disease (39.1% *vs*. 15.5%, respectively; 95% CI 0.02 to 0.45; *p* = 0.03).

Thirty-one patients had undergone tracheostomy before surgery. Nine of them had PCF after laryngectomy. Tracheostomy did not increase the risk of PCF (*p* = 0.22). However, 40.9% of the patients who had undergone tracheostomy before surgery had fistulae, against 21.1% of the subjects not submitted to tracheostomy. Age, type of neck dissection and pharyngeal closure were not statistically significant.

Our patients had PCF from days 3 and 8 after surgery. Mean length of hospitalization was 12.8 days, against three days for the non-PCF group.

## DISCUSSION

The approach chosen to treat PCF may considerably increase the length of hospitalization and cost of care, in addition to delaying the start of adjuvant radiotherapy when indicated. PCF is a complication that usually occurs shortly after total laryngectomy.

Our patients had PCF from three to eight days after surgery. Mean length of hospitalization was 12.8 days, against three days for the non-PCF group. Patients were discharged as early as possible, even when they had pharyngeal fistula (PF), and kept in home care.

In terms of primary tumor site, patients with supraglottic tumors were at a higher risk for PCF, as in these cases subjects may be submitted to large resections of the pharyngeal mucosa and the stitches might be under significant strain^5^. In another series, only the patients submitted to combined partial pha-ryngectomy and total laryngectomy procedures were statistically at risk of PCF^2^. In our series, the higher prevalence of glottic tumors did not allow further comparison. Additionally, patients who required more extensive resection of the pharyngeal mucosa had their reconstructions done with flaps, which excluded them from the study.

Some authors have found that neck dissection done concurrently to total laryngectomy increased the incidence of PCF^5^, ^11^. This variable was not statistically significant in our sample (*p* = 0.81).

There is disagreement in the literature in regards to the impact of previous radiotherapy. Some authors found it not to be statistically significant^2^, ^4^, ^5^, while others found relevant correlations^8^, ^12^. PCF has been reported to occur earlier in previously irradiated patients^5^. There is a 2% to 3% chance of PCF when total laryngectomy is performed as the primary treatment, but incidence rates soar to 10% to 12% when it is done after radiotherapy, and increase to even higher levels when other neck procedures and/or more extensive surgery are performed^10^. Radiotherapy dosages greater than 5,000 cGy have been reported to increase PCF incidence rates^13^. Another study reported rates of 80% among surgery patients given 6,800 to 7,200 cGy^14^.

Organ preservation protocols have been considered as an important option in the treatment of advanced laryngeal cancer. However, it is yet unknown whether the addition of chemotherapy and radiotherapy increases the chance of postoperative complications in the context of salvage total laryngectomy. In a prospective randomized trial 517 patients were divided into three arms: 1) induction chemotherapy followed by radiotherapy; 2) chemoradiotherapy; and 3) radiotherapy alone. PCF incidence rates were lower in the third arm (15%) and higher in the second (30%)^15^. Our study featured a group with previous chemoradiotherapy (organ preservation protocol) and salvage total laryngectomy for relapsing and residual disease (n = 32) and a group with laryngectomy as the primary therapy (n = 62), but their PCF incidence rates were not statistically different (*p* = 0.54).

Tracheostomy did not increase the risk of PCF (*p* = 0.22). However, 40.9% of the patients who had undergone tracheostomy before surgery had fistulae, against 21.1% of the subjects not submitted to tracheostomy. It is possible that a larger sample of patients could elicit the significance of this trend. A study done with 55 patients found significantly higher rates of PCF in patients previously submitted to tracheostomy (60% *vs*. 8%) - *p* = 0.012^16^. The possible explanations for this finding include more advanced disease, fibrosis, and local contamination by tracheal secretion. Indeed, tracheostomy is frequently offered to patients manifesting respiratory insufficiency on their first visit with the specialized physician, a sign of advanced primary tumor^17^. And the T-stage was considered in our study as a statistically significant factor for the occurrence of PCF.

Blood transfusions were not considered in the assessment done in this study, as they were rarely offered during surgery. But this is a controversial topic in the literature. While one study reports a 28% incidence rate of PCF in patients given blood product transfusions against a 7% rate for patients not given transfusion^18^, other authors failed to find such correlation^1^, ^19^. Transfusion is more indicated in larger procedures, usually associated with more advanced disease.

Pharyngeal closure using linear staplers during total laryngectomy has become more popular in the last three decades^20^. Manual stitches take longer, lead to more necrosis of the pharyngeal mucosa, and contamination of the surgery site with saliva. Additionally, there is a weak spot in manual laryngeal closure: the junction point in the T-shaped stitches. This weak spot is not produced when a stapler is used. Stapling reduces the rate of PCF, as revealed in a meta-analysis^21^.

Another factor correlated to early PCF is histological infiltration in the surgical margins of the tumor - 11% negative *vs*. 38% positive^9^. This variable was not considered in our study, as intraoperative histopathology testing of the frozen margins removed from the patient was done after the removal of the surgical specimen. This was done as a safety measurement. On the other hand, part of the tumors, particularly the ones removed in stapler-assisted pharyngeal closure, are endolaryngeal, in which case broader resection margins are available.

Salivary fistulae usually occur between five to seven days after surgery. Oral feeding is normally reintroduced after this period^22^. The nasoenteral feeding tube is usually removed ten days after surgery in patients without signs of PCF. Studies have recently discussed the early reintroduction of oral feeding in total laryngectomy patients as a factor that does not increase the risk of complication. A case-control study revealed that early peroral feeding was not considered as a risk factor^23^.

## CONCLUSION

Patients with advanced primary tumors are at a higher risk of PCF.
